# Abolition of the Practice of Variolous Inoculation

**Published:** 1840-07

**Authors:** 


### ABOLITION OF THE PRACTICE OF VARIOLOUS INOCULATION.

Science and humanity have gained a great triumph by the recent decision of the House of Commons that henceforth it shall be illegal to inoculate for small-pox. For this decision the profession and the public are indebted to the persevering exertions of Mr. Wakley; and if the resolution of the House of Commons is finally passed into a law, as we hope and trust it will be, this gentleman will have just cause to pride himself on being the means of conferring an inestimable boon on his country. We greatly regret that the whole subject of vaccination has not been legislated on in a more systematic and scientific manner; but we are very thankful for the great benefit now acquired; and we hope that when the profession has obtained a proper organization and possesses a council that really represents it, the whole superintendence of this most important matter will be transferred to a scientific board.

